# Acute Effects of Aerobic Exercise on Muscle Strength and Power in Trained Male Individuals: A Systematic Review with Meta-analysis

**DOI:** 10.1007/s40279-021-01615-6

**Published:** 2021-12-08

**Authors:** Adrian Markov, Helmi Chaabene, Lukas Hauser, Sebastian Behm, Wilhelm Bloch, Christian Puta, Urs Granacher

**Affiliations:** 1 Division of Training and Movement Sciences, Research Focus Cognition Sciences, Faculty of Human Sciences, University of Potsdam, Am Neuen Palais 10, Bldg. 12, 14469 Potsdam, Germany; 2 Department of Molecular and Cellular Sport Medicine, German Sport University, Cologne, Germany; 3 Department of Sports Medicine and Health Promotion, Friedrich-Schiller-University Jena, Jena, Germany; 4 Faculty of Human Sciences, University of Potsdam, Potsdam, Germany

## Abstract

**Background:**

Concurrent training can be an effective and time-efficient method to improve both muscle strength and aerobic capacity. A major challenge with concurrent training is how to adequately combine and sequence strength exercise and aerobic exercise to avoid interference effects. This is particularly relevant for athletes.

**Objective:**

We aimed to examine the acute effects of aerobic exercise on subsequent measures of muscle strength and power in trained male individuals.

**Design:**

We performed a systematic review with meta-analysis.

**Data Sources:**

Systematic literature searches in the electronic databases PubMed, Web of Science, and Google Scholar were conducted up to July 2021.

**Eligibility Criteria for Selecting Studies:**

Studies were included that applied a within-group repeated-measures design and examined the acute effects of aerobic exercise (i.e., running, cycling exercise) on subsequent measures of lower limb muscle strength (e.g., maximal isometric force of the knee extensors) and/or proxies of lower limb muscle power (e.g., countermovement jump height) in trained individuals.

**Results:**

Fifteen studies met the inclusion criteria. Aerobic exercise resulted in moderate declines in muscle strength (standardized mean difference [SMD] = 0.79; *p* = 0.003). Low-intensity aerobic exercise did not moderate effects on muscle strength (SMD = 0.65; *p* = 0.157) while moderate-to-high intensity aerobic exercise resulted in moderate declines in muscle strength (SMD = 0.65; *p* = 0.020). However, the difference between subgroups was not statistically significant (*p* = 0.979). Regarding aerobic exercise duration, large declines in muscle strength were found after > 30 min (SMD = 1.02; *p* = 0.049) while ≤ 30 min of aerobic exercise induced moderate declines in muscle strength (SMD = 0.59; *p* = 0.013). The subgroup difference was not statistically significant (*p* = 0.204). Cycling exercise resulted in significantly larger decrements in muscle strength (SMD = 0.79; *p* = 0.002) compared with running (SMD = 0.28; *p* = 0.035). The difference between subgroups was statistically significant (*p* < 0.0001). For muscle power, aerobic exercise did not result in any statistically significant changes (SMD = 0.04; *p* = 0.846).

**Conclusions:**

Aerobic exercise induced moderate declines in measures of muscle strength with no statistically significant effects on proxies of muscle power in trained male individuals. It appears that higher compared with lower intensity as well as longer compared with shorter aerobic exercise duration exacerbate acute declines in muscle strength. Our results provide evidence for acute interference effects when aerobic exercies is performed before strength exercises. These findings may help practitioners to better prescribe single training sessions, particularly if environmental and/or infrastructural reasons (e.g., availability of training facilities) do not allow the application of strength training before aerobic exercise.

## Key Points

**Table:** 

Aerobic exercise resulted in acute moderate declines in measures of muscle strength but not power in trained male individuals.
There was a negative influence of prior moderate-to-high intensity, as well as longer aerobic exercise durations (i.e., > 30 min) on muscle strength in trained male individuals. Low-intensity and short duration (i.e., ≤ 30 min) aerobic exercise appears not to compromise strength performance.
Cycling compared with running exercise causes larger decrements in lower limb muscle strength.

## Introduction

Many sports require the simultaneous development of muscle strength and aerobic capacity to successfully perform in competition [1–3]. Concurrent training (CT), which is the combination of strength and endurance training, can be an effective and time-efficient method to improve both muscle strength and aerobic capacity [4, 5]. The long-term effects of CT on measures of muscle strength and muscle power are well established in the literature [6–10]. A major challenge within CT is how to adequately combine strength and endurance training to avoid interference effects [11–13]. In this context, interference has been reported if CT was contrasted with single-mode strength training. The results showed that the adaptive potential of CT to improve muscle strength is attenuated compared with single-mode strength training [7, 9, 13–15]. In general, the interference may occur in elite and recreational athletes if they exercise at high training volumes. However, the training volume of recreational athletes is usually low to moderate, which prevents interference if the training is appropriately prescribed. In contrast, elite athletes in sports that demand both high levels of muscle strength and aerobic capacity exercise up to 25 h per week [16], which increases the likelihood of experiencing interference effects due to timely proximity of strength and endurance training.

This raises the question with regard to the most effective CT sequence to minimize interference. Murlasits and colleagues [17] conducted a systematic review with meta-analysis and examined the long-term effects of intra-session exercise sequence during CT on lower body muscle strength and maximal aerobic capacity in healthy individuals aged 14–66 years. These authors reported that the sequence strength exercises (SE) before aerobic exercises (AE) is more effective to improve muscle strength than AE before SE. Using a similar meta-analytical approach, Eddens and colleagues [18] confirmed the findings of Murlasits et al. [17] for lower limb maximal strength in healthy individuals aged 18–65 years.

While the long-term effects of intra-session exercise sequencing during CT on muscle strength are well established, little is known on the acute effects of exercise sequencing during CT. In this context, the term ‘acute’ refers to the impact of the sequence (SE before AE or vice versa) on muscle strength and power after a single training session. This is of particular relevance for elite athletes who realize up to four training sessions per day [16]. As such, it is not always possible to follow the recommended SE-AE sequence [17, 18] in every single training session because of the training schedule or the availability of training facilities.

Of note, the available studies on the acute effects of AE on measures of muscle strength and power have shown inconsistent findings. More specifically, while some studies reported attenuated strength and power outcomes [19–21], others showed post-activation performance enhancement (PAPE) most likely triggered by the previous AE [22–24]. For example, Lepers et al. [25] studied the acute effects of two different cycling modalities (i.e., constant and variable power output) on measures of muscle strength in trained male triathletes aged 33 years. Training was conducted at ~ 63–86% of the maximal aerobic power (i.e., highest power in Watts across 2 min during a continuous incremental cycling test until exhaustion) for 30 min. The results revealed a decrease in knee extensor isometric maximal voluntary contraction (IMVC) [∆11%] following both cycling modalities. In another study, Wilhem and colleagues [26] examined the effects of 30 min of cycling or running at 75–85% of the respiratory compensation point on measures of muscle strength and power in recreationally trained male individuals aged 23 years. These authors could not find any significant performance changes after both protocols. Additionally, in trained long-distance runners aged 24 years, Vuorimaa and colleagues [27] reported improved vertical jump performance (∆10–15%) following 40 min of continuous or intermittent (i.e., 2-min run/2-min rest) running at 80% and 100% of the maximum running speed achieved during a graded maximal oxygen consumption (*V̇*O_2max_) test, respectively. The observed discrepancy in the literature is most likely due to differences in the participants’ training status [12], the type of task used to quantify fatigue [12], AE-related neuromuscular fatigue [28, 29], and/or PAPE [30]. Neuromuscular fatigue appears to be a major candidate responsible for the observed AE-related performance declines [31–33]. For this systematic review, we have defined neuromuscular fatigue as a reduction in muscle force and power in response to sustained contractile activity [28, 29]. There is evidence that AE intensity and volume (i.e., duration) are key moderators for AE-induced neuromuscular fatigue [34, 35]. In contrast, PAPE refers to gains in muscle power, speed, and maximal strength following conditioning contractions [30]. Other important factors that might (partly) explain the conflicting findings in the literature are the complexity of the underpinning mechanisms of adaptations as well as factors such as the rest between AE and SE and type of SE/AE [4, 12, 36].

Accordingly, it seems necessary to perform a systematic review of the literature on the acute effects of AE on measures of muscle strength and power in trained individuals. Therefore, this systematic review with meta-analysis aimed at (1) examining the acute effects of AE on subsequent measures of muscle strength and power in trained male individuals and (2) investigating the influence of potential moderating factors such as AE intensity, duration, and type (i.e., low vs moderate-to-high intensity AE, ≤ 30 min vs > 30 min AE, and cycling vs running exercise) on muscle strength and power. With reference to the relevant literature [19–21, 25], we hypothesized that findings from the included AE studies would show declines in measures of muscle strength and power due to AE-related neuromuscular fatigue. We further expected that the magnitude of declines in measures of muscle strength and power would be moderated by different exercise modalities (e.g., AE intensity and duration) [26, 37] and types (i.e., cycling vs running) [12].

## Methods

This meta-analysis was conducted according to the Preferred Recording Items for Systematic Review and Meta-analysis (PRISMA) statements [38].

### Systematic Literature Search

**Fig. 1:**
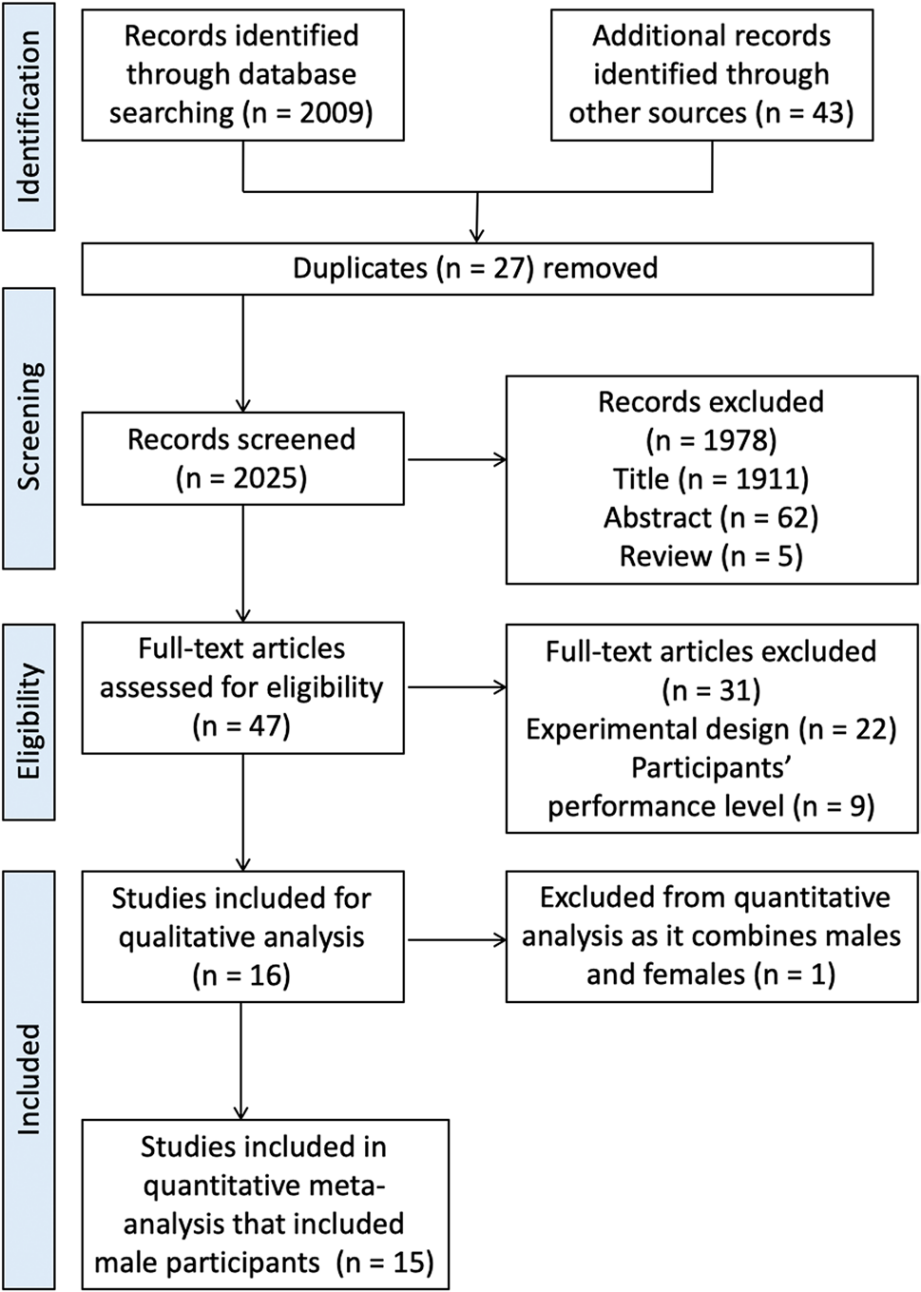
Flow chart illustrating the search and selection process of this systematic review

A systematic literature search was conducted in the electronic databases PubMed, Web of Science, and Google Scholar up to July 2021. Keywords were collected through expert opinion, literature review, and controlled vocabulary (e.g., Medical Subject Headings [MeSH]). The following Boolean search syntax is an example of a PubMed search: “(strength OR resistance OR endurance OR aerobic OR concurrent OR running OR cycling OR rowing OR swimming) AND (training OR exercise) AND (order OR intra-session OR sequence OR within-session OR “same day” OR “separate day” OR acute OR short-term) AND (combined OR combination OR additional OR subsequent) NOT (patients OR elderly OR cancer OR diabetes OR injury OR protein OR stretching OR obese OR blood OR diet OR rat OR cognitive OR stroke)”. Where applicable, filters were applied for the type of article (e.g., no reviews). The search results were independently screened for titles, abstracts, and full texts by two authors (AM, SB). In addition, reference lists of the included studies were screened for more compatible studies. An overview of the search process is displayed in Fig. 1.

### Inclusion and Exclusion Criteria

A PICOS (participants, intervention, comparator, study outcomes, and study design) approach was used to rate studies for eligibility [38]. Specifically, the following inclusion criteria were defined a priori: (1) participants: trained youth and young adults (2) intervention: AE (i.e., running, cycling) (3) comparator: post-test measure within ≤ 15 min after AE (4) study outcomes: muscle strength (e.g., maximal isometric force of the knee extensors) and/or power, (e.g., countermovement jump height), and (5) study design: within-group repeated-measures design. Of note, there is no well-accepted definition of the term “trained,” especially in the context of CT [39]. However, with reference to the included studies and based on the definition provided by MacMahon and Parrington [40], we consider trained individuals as athletes actively engaging in sports training, where the main motivation or goal is to improve sport-specific skills, performance, or results (technical, physical, or tactical) for competition. In this context, AE is an umbrella term for all types of physical exercise to improve cardiorespiratory fitness (e.g., oxygen transport and utilization systems) [41]. We excluded studies involving individuals with pre-existing health problems (e.g., diabetes mellitus, hypertension) or with no training background, lack of pre-AE and post-AE measures, and studies not written in English.

### Data Extraction

Studies were coded for the variables displayed in Table 1. When multiple tests were used for the same outcome measure, the most accurate and frequently used protocols were selected based on expert opinion (AM, HC, LH, and UG) (Table 1). Two authors (AM and SB) independently extracted data from the included studies in a standardized template created with Microsoft Excel (version 16.16.27). In cases of disagreement regarding data extraction and study eligibility, another co-author (HC) was consulted for clarification. To compute effect sizes, pre-test and post-test means and standard deviations for measures of muscle strength and power were used. The characteristics of the included studies are displayed in Table 2. Of note, intermittent and continuous AE protocols were treated in the same way in terms of duration. More specifically, the cumulative duration of the work intervals, including the respective recovery periods between intervals, were considered for intermittent protocols. In the case of missing data, authors were contacted and kindly asked to provide the data. If the authors did not respond and the data were displayed graphically, we used a software tool (GetData Graph Digitizer; http://www.getdata-graph-digitizer.com) [42], which has been shown to be accurate and precise [43], to extract data from graphs.

**Table 1:** Testing protocols across the different measures of physical fitness considered for statistical analyses

Outcome categories	Ranking
Muscle strength	•Maximal isometric force of the knee extensors •Maximal isokinetic torque of the knee extensors •One-maximum repetition knee extensors (leg press)
Muscle power	•CMJ height •CMJ peak power

*CMJ* counter movement jump

**Table 2:** Study characteristics

References	NP	Age (mean ± SD)	Sport discipline	Exercise type	Session duration (min)	Exercise intensity	Exercise mode	Post-measure (min)	Muscular fitness component	Parameter measured	Main result	Study quality
Bentley et al. [53]	10	25 ± 7	Triathletes	Cycling	> 30	Moderate-high	Continuous + intermittent	+ 10	MS	Isometric knee extension (N)	↓ (∆12%)	7.3
Boullosa et al. [58]	12	23 ± 3	Runners	Running	> 30	Low	Continuous	+ 2	MP	CMJ height (cm)	↑ (∆13%)	8.5
Boullosa et al. [22]	22	24 ± 5	Runners/triathletes	Running	≤ 30	Moderate-high	Continuous	+ 0	MP	CMJ height (cm)	↑ (∆4%)	8.5
Garcia-Pinillos et al. [23]	30	28 ± 8	Runners	Running	> 30	Moderate-high	Continuous	+ 0	MP	CMJ height (cm)	↑ (∆14%)	9.3
Gomez et al. [54]	10	23 ± 3	Runners	Running	> 30	Moderate-high	Continuous	+ 15	MS	Isokinetic peak torque extension at 30° (Nm)	↓ (∆4%)	10.5
Gomez et al. [54]	10	23 ± 3	Runners	Running	> 30	Moderate-high	Continuous	+ 15	MP	CMJ peak power (W)	↑ (∆1%)	11
Johnston et al. [59]	15	21 ± 1	Rugby	Running	> 30	Moderate-high	Continuous	+ 0	MP	CMJ height (cm)	↓ (∆9%)	11
Lattier et al. [34]	8	25 ± 4	N.A	Running	≤ 30	Moderate-high	Continuous	+ 0	MS	Isometric knee extension at 80° and 85° (Nm)	↓ (∆8%)	8.8
Lattore-Roman et al. [24]	16	30 ± 7	Runners	Running	> 30	Moderate-high	Continuous	+ 0	MP	CMJ height (cm)	↑ (∆2%)	8.3
Lepers et al. [35]	8	26 ± 4	Triathletes	Cycling	> 30	Low	Continuous	+ 0	MS	Isometric knee extension at 60° (Nm)	↓ (∆13%)	7.8
Lepers et al. [25]	8	33 ± 5	Triathletes	Cycling	≤ 30	Low	Continuous	+ 0	MS	Isometric knee extension (Nm)	↓ (∆11%)	7.3
Sparkes et al. [60]	14	22 ± 3	Soccer	SsSG	> 30	N.A	Intermittent	+ 0	MP	CMJ height (cm)	↓ (∆2%)	9.5
Sparkes et al. [61]	12	21 ± 2	Soccer	SsSG	> 30	N.A	Intermittent	+ 0	MP	CMJ height (cm)	↓ (∆1%)	9.5
Thomas et al. [57]	13	31 ± 8	Cycling	Cycling	≤ 30	Moderate-high	Continuous	+ 3	MS	Isometric knee extension at 90° (N)	↓ (∆16%)	9
Thomas et al. [56]	12	28 ± 8	Cycling	Cycling	≤ 30	Moderate-high	Continuous	+ 3	MS	Isometric knee extension at 90° (N)	↓ (∆16%)	8
Thomas et al. [55]	15	21 ± 1	Soccer	SSM	> 30	N.A	Intermittent	+ 0	MS	Isometric knee extension at 90° (N)	↓ (∆45%)	8
Thomas et al. [55]	15	21 ± 1	Soccer	SSM	> 30	N.A	Intermittent	+ 0	MP	CMJ height (cm)	↓ (∆13%)	8
Vuorimaa et al. [27]	22	19–33	Runners	Running	> 30	Moderate-high	Intermittent	+ 0	MP	CMJ height (cm)	↑ (∆10%)	8

*CMJ* counter movement jump, *min* minutes, *MS* muscle strength, *N* Newton, *N.A.* not applicable, *Nm* Newton meter, *NP* number of participants, *MP* muscle power, *W* Watts, *SSM* simulated soccer match, *SsSG* small sided soccer game, ↑ (∆%) indicates an increase in measure of muscle strength/power, ↓ (∆%) indicates a decrease in measure of muscle strength/power

### Risk of Bias Assessment

The methodological quality of the included studies is summarized in Table 2. The risk of bias assessment was conducted independently by two authors (AM, LH), using the quality appraisal tool developed by Galna et al. [44]. The applied quality appraisal tool focuses on internal and external validity as well as the reproducibility of the study. It includes 14 items and allows a rating between 0 and 1, with 1 representing the maximum value for each of the items. There is no particular classification (e.g., low, acceptable, high) foreseen with this rating system. The higher the total score, the better the quality of the respective study. Of note, the highest possible score is 14. Total scores ranged between 7 and 11 (Table 2). In the case of disagreement, another co-author (HC) was consulted for clarification.

### Statistical Analyses

The effects of AE on subsequent measures of muscle strength and power were examined by calculating standardized mean differences (SMDs) for pre-AE and post-AE measures of the respective studies. To estimate the overall effect of AE on subsequent measures of muscle strength and power, we pooled effect sizes using a random-effects pooling model approach with the Sidik-Jonkman estimator method and Hartung-Knapp adjustment [45] using the packages *“meta”* [46] and *“metafor”* [47]. In addition, independent subgroup analyses were calculated for the exercise modalities AE intensity (low vs moderate to high), AE duration (≤ 30 min vs > 30 min), and AE type (running vs cycling exercise). Of note, because of a limited number of studies per group, subgroup analyses were calculated only for measures of muscle strength and not power. Based on the guidelines proposed by the American College of Sports Medicine [48], we classified AE intensity as low (< 70% *V̇*O_2max_; < critical power; < peak aerobic power; < anaerobic or lactate threshold), or moderate to high (> 70% *V̇*O_2max_ or maximal heart rate; > critical power; > anaerobic or lactate threshold; rate of perceived exertion ≥ 15). Critical power was defined as the highest power output in Watts that can be sustained for a given period of time [49]. Standardized mean differences were interpreted according to Cohen [50] as “trivial” (< 0.2), “small” (0.2 ≤ SMD < 0.5), “moderate” (0.5 ≤ SMD < 0.8), or “large” (SMD ≥ 0.8). *I*^2^ statistics were used to examine between-study heterogeneity. According to Higgins et al. [51], heterogeneity in the form of *I*^2^ statistics was interpreted as “low” (25%), “moderate” (50%), and “high” (75%). The level of significance was set at *p* < 0.05. All analyses were conducted using R (version 4.0.2, 2020) [52] and validated open-source packages [47].

## Results

### Study Characteristics

Our systematic search identified 2052 potential articles (Fig. 1). After screening for titles, abstracts, and full texts, 15 studies were considered eligible with a total of 197 participants. The age range within the included studies was 18–42 years. Eight studies examined AE effects on measures of muscle strength [25, 34, 35, 53–57], and another nine studies reported AE effects on proxies of muscle power [23, 24, 27, 54, 55, 58–61]. Running-based AE was examined in seven studies [23, 24, 27, 34, 54, 58, 59] while cycling exercises were applied in five studies [25, 35, 53, 56, 57]. Three studies scrutinized the effects of sport-specific AE (i.e., small-sided soccer games) [55, 60, 61]. Likewise, two studies applied low-intensity AE [25, 35] while 11 studies used moderate-to-high intensity AE protocols [23, 24, 27, 34, 53, 54, 56, 57, 59, 62, 63]. However, three studies [55, 60, 61] did not report any information on exercise intensity. In terms of AE duration, four studies used protocols lasting ≤ 30 min [25, 34, 56, 57] and 11 studies applied protocols > 30 min [23, 24, 27, 35, 53–55, 58–61]. Originally 16 studies matched the inclusion criteria. Because of the fact that only one paper considered male and female individuals [22], this study was excluded for the quantitative meta-analysis to ensure consistency throughout the data.

### Acute Effects of Aerobic Exercise on Measures of Muscle Strength and Power

**Fig. 2:**
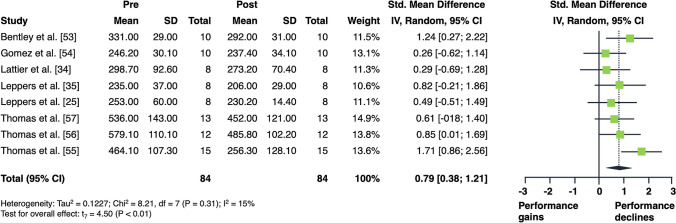
Forest plot for the overall effect of aerobic exercise on subsequent measures of muscle strength

**Fig. 3:**
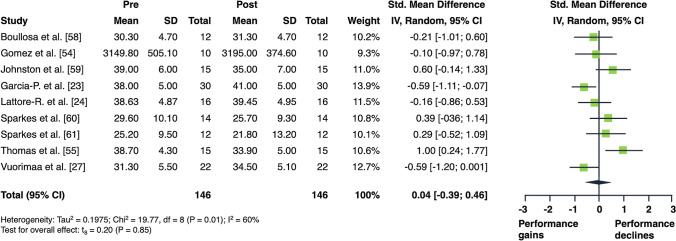
Forest plot for the overall effect of aerobic exercise on subsequent measures of muscle power

The overall acute effects of AE on measures of lower limb muscle strength and power are illustrated in Figs. 2 and 3. Aerobic exercise resulted in moderate declines in measures of muscle strength (SMD = 0.79 [95% confidence interval (CI) 0.38 to 1.21]; *p* = 0.003; *I*^2^ = 15%, eight studies, Fig. 2). Regarding muscle power, AE did not produce any statistically significant changes (SMD = 0.04 [95% CI − 0.39 to 0.46]; *p* = 0.846; *I*^2^ = 60%, nine studies, Fig. 3).

### Results of the Subgroup Analyses for Measures of Muscle Strength

Results of the subgroup analyses are displayed in Table 3. Low-intensity AE did not cause statistically significant effects on muscle strength (SMD = 0.65 [95% CI − 1.45 to 2.75], *p* = 0.157; *I*^2^ = 0%, two studies) while moderate-to-high intensity AE resulted in moderate declines in muscle strength (SMD = 0.65 [95% CI 0.16–1.13], *p* = 0.020; *I*^2^ = 0%, five studies). However, the difference between subgroups was not statistically significant (*p* = 0.979). Regarding AE duration, large declines in muscle strength were found after > 30 min (SMD = 1.02 [95% CI 0.01–2.03], *p* = 0.049; *I*^2^ = 47.4%, four studies) while ≤ 30 min of AE induced moderate decrements in muscle strength (SMD = 0.59 [95% CI 0.23–0.95], *p* = 0.013; *I*^2^ = 0%, four studies). Nevertheless, no statistically between-subgroup differences were observed (*p* = 0.204). Running exercise resulted in small declines in muscle strength (SMD = 0.28 [95% CI 0.08–0.47], *p* = 0.035; *I*^2^ = 0%, two studies) while cycling exercise produced moderate declines in muscle strength (SMD = 0.79 [95% CI 0.45–1.13], *p* = 0.002; *I*^2^ = 0%, five studies). Of note, the difference between subgroups was statistically significant (*p* < 0.0001).

**Table 3:** Subgroup analysis for measures of muscle strength

Subgroup	Studies (*N*)	Participants (*N*)		Estimated effect size Mean (95% CI)	Within-group *p*-value	Between subgroup *p-*value	Effect descriptor
AE intensity							
Low	2		16	0.65 (− 1.45 to 2.75)	0.157	0.979	
Moderate to high	5		53	0.65 (0.16–1.13)	0.020		Medium
AE type							
Running	2		18	0.28 (0.08–0.47)	0.035	< 0.001	Small
Cycling	5		51	0.79 (0.45–1.13)	0.002		Medium
AE duration							
≤ 30 min	4		41	0.59 (0.23–0.95)	0.013	0.204	Medium
> 30 min	4		43	1.02 (0.01–2.03)	0.049		Large

*AE* aerobic exercise, *CI* confidence interval

## Discussion

This is the first systematic review and meta-analysis to examine the acute effects of AE on measures of muscle strength and power in trained male individuals. The main findings of this systematic review with meta-analysis indicated that (1) AE resulted in acute moderate declines in subsequent (i.e., ≤ 15 min) measures of lower limb muscle strength with no statistically significant effects on proxies of lower limb muscle power, (2) moderate-to-high intensity AE induced a moderate decline in muscle strength with no statistically significant effects of low-intensity AE, (3) AE lasting > 30 min resulted in a large decline in muscle strength, whereas ≤ 30 min induced moderate declines, and (4) cycling exercise caused significantly larger decrements in muscle strength compared with running exercise.

### Main Effects of Aerobic Exercise on Subsequent Measures of Lower Limb Muscle Strength and Power

The main effects of this study indicated that AE resulted in declines in muscle strength (SMD = 0.79) but not power (SMD = 0.04) in trained male individuals. In general, these findings are in agreement with the literature [34, 53]. Bentley et al. [53] studied the effects of AE until exhaustion on knee extensor IMVC and muscle activation in trained male athletes aged 25 years. They reported that AE resulted in significant IMVC declines immediately post-exercise (∆12%) and after 6 h (∆6%) of rest. In addition, the authors showed that IMVC declines were related to reduced electromyographic activity and twitch torque, which is indicative of both central and peripheral mechanisms of neuromuscular fatigue [53]. Furthermore, Lattier et al. [34] examined the effects of high-intensity intermittent exercise (10 runs of 1 min each at 120% of maximal aerobic velocity) on subsequent knee extensor IMVC in well-trained male individuals aged 25 years. They reported an IMVC decline immediately (∆8%) and 65 min (∆6%) after AE. The same authors attributed the attenuation in IMVC to altered excitation–contraction coupling (decreased twitch contractile properties) and decreased maximal activation of the muscle. According to Lattier et al. [34], high-intensity intermittent exercise appears to induce both peripheral and central fatigue. In this context, the available literature is inconsistent. For example, Burnley and Jones [64] emphasized that the origin of neuromuscular fatigue depends on exercise intensity while Bishop [65] reported that fatigue following intermittent sprint exercise is primarily caused by peripheral mechanisms. Overall, there is ample evidence that AE has acute negative effects on measures of muscle strength [25, 66]. Of note, Lattier et al. [34] and Bentley et al. [53] reported that declines in IMVC last longer. Nevertheless, it should be highlighted that because of a lack of data, the quantitative analyses of this systematic review with meta-analysis solely considered measures of muscle strength within a time frame of ≤ 15 min after AE.

Unlike muscle strength, our findings indicated no significant effects of AE on subsequent measures of muscle power (SMD = 0.04). In previous studies, effects of an all-out 10-km running competition on subsequent measures of muscle power (i.e., peak vertical jump power) were examined in male endurance athletes aged 18–26 years [54]. The main results indicated no changes in peak vertical jump power immediately after the 10-km race. These authors speculated that motor unit recruitment during a 10-km race does not involve fast-twitch muscle fibers, which are needed to produce high levels of muscle power [54]. Boullosa et al. [58] examined the effects of two endurance tasks performed until voluntary exhaustion (i.e., ‘‘Université de Montréal Track Test’’ and the time to exhaustion at maximal aerobic speed) on vertical jump performance (i.e., countermovement jump height) in well-trained male endurance runners aged 23 years. The authors reported an increase in vertical jump height 2 min after the two running protocols (∆13% and ∆4%, respectively), an observation attributed to PAPE [30]. Other studies have also shown AE-related PAPE on measures of muscle power [23, 27]. In this context, it is worth mentioning that PAPE is time dependent (up to 10 min) but also moderated by the performance level of the respective athlete [67, 68]. Furthermore, there are studies that reported a decline in vertical jump performance following AE [55, 59]. For example, Johnston et al. [59] examined the effects of a maximum speed running session on vertical jump height in professional rugby players aged 21 years and found a significant decrease immediately after AE (∆9%), which returned to baseline 2 h post-AE. Similar results were reported by Thomas et al. [55]. These authors [55] examined the effects of a simulated soccer match on measures of muscle power in professional players aged 21 years and reported a significant decrease in vertical jump height immediately after (∆13%), which lasted for up to 72 h (∆5%). Taken together, these contradictory findings highlight the complex nature of neuromuscular fatigue. Indeed, our results suggest that neuromuscular fatigue is influenced by a large number of factors (e.g., individual training history or type of task applied).

Overall, our findings support the hypothesis that AE impairs subsequent measures of muscle strength [35, 57]. As to the underlying mechanisms, there is evidence that AE causes central (impaired neural drive) and peripheral (impaired excitation–contraction coupling) fatigue [34, 53] which ultimately results in muscle strength declines. Moreover, metabolic factors triggered by the AE seem to play a major role. Findings from different studies indicate that AE results in a decline in Ca^2+^ sensitivity, which may again inhibit the conversion of electrical stimuli to mechanical responses [69–71]. Results from animal and human studies [72] showed that exercise (e.g., cycling at 70% of V̇O_2max_) induces an increase in ryanodine receptor 1 phosphorylation that impairs myofibrillar Ca^2+^ processing and consequently muscle function. Additional factors such as metabolic acidosis (e.g., pH reduction and/or Pi accumulation) and impaired action potential (e.g., extracellular K^+^ accumulation) appear to be fostered by AE [69, 71]. Accordingly, it was reported that high AE volume increases extracellular K^+^ accumulation [73, 74], altering sarcolemmal excitability [75]. Thereby, it seems that the above described physiological factors (e.g., muscle activation, defect in Ca^2+^release, Pi accumulation) are moderated by AE modalities such as volume and intensity [12, 37].

Findings from this systematic review with meta-analysis are in line with the results from previous meta-analyses [17, 18] on chronic AE-SE sequencing effects. The existing data indicate that SE before AE, but not AE before SE, is more effective for improving muscle strength. In contrast, several studies [76–78] showed that high-intensity interval AE but also continuous AE performed prior to SE do not inhibit muscle protein synthesis. The gap between these findings may result from a multitude of possible CT modalities. Alternatively, muscular hypertrophy may not have been a major determinant of the training-induced strength improvements following SE-AE sequencing reported in previous studies [17, 18]. Future studies should elucidate the underlying physiological mechanisms following different CT modalities. Further, our findings suggest that the capacity to generate maximal muscular power after AE is less affected compared with muscle strength. For instance, Latorre-Román et al. [24] examined the effects of running exercise (i.e., 4 × 3 × 400 m) in trained runners and found no statistically significant change in vertical jump performance after AE. The production of muscle force can be regulated through motor unit recruitment, firing frequency, and synchronization [79]. Firing frequency appears to be a major mechanism that enables the muscle to rapidly generate force. Accordingly, firing frequency represents a major neural factor to regulate muscle power [80, 81]. Even though highly speculative, it can be argued that the applied AE protocols may have affected peripheral neuromuscular mechanisms rather than central mechanisms, which is why we found declines in muscle strength but not power. Given that this is a systematic review of the literature and not original research using electrophysiological testing, future studies are needed to verify this hypothesis.

### Subgroup Analyses

Our results suggest that moderate-to-high intensity (SMD = 0.68) but not low-intensity AE causes a decline in muscle strength. However, it should be mentioned that the observed differences between subgroups were not statistically significant and that only two studies were included that examined low-intensity training. Therefore, these results should be interpreted with caution. Our findings are in line with the available literature. For example, Lepers et al. [25] reported that male triathletes who cycled at an intensity of 75% of their maximal aerobic power (i.e., highest power in Watts across 2 min during a continuous incremental cycling test until exhaustion) experienced a 9% decline in muscle strength. In addition, the same authors showed that cycling at 80–90% resulted in a 13% decline in maximal aerobic power. In terms of AE duration, our findings showed larger decrements for AE lasting > 30 min compared with ≤ 30 min (SMD = 0.97 vs SMD = 0.72, respectively). However, the difference was not statistically significant. Lepers et al. [35] investigated the effects of 2 h of cycling at 65% of maximal aerobic power in trained cyclists and found a significant reduction in maximal isometric force of the knee extensors (∆13%). In addition, Lattier et al. [34] investigated the effects of high-intensity uphill running (10 runs of 1 min each at 120% maximal aerobic velocity) in trained individuals and reported a significant decrease in isometric force of the knee extensors after AE (∆8%). Regarding the underlying mechanisms, there is evidence that muscle glycogen depletion increases progressively, either with extended exercise duration and/or increased exercise intensity [82]. Moreover, earlier studies [35, 56, 57] reported an intensity-dependent and duration-dependent magnitude of peripheral and central fatigue.

The current findings suggest an AE-related effect that is specific to the applied AE type. More specifically, the results showed that cycling resulted in significantly larger decrements in muscle strength (SMD = 0.79) compared with running exercise (SMD = 0.28). There is evidence that cycling and running are associated with mitigated strength adaptations following CT compared with single-mode strength training [12]. However, cycling exercise has been associated with smaller interference effects compared with running exercise [83]. Amongst others, this can be attributed to eccentric muscle actions during running, which may increase muscle damage and therefore contribute to declines in measures of muscle strength and power [84]. Our results are contradictory to the existing literature and suggest that, for trained individuals, running exercise could be more appropriate to avoid neuromuscular fatigue and declines in muscle strength when AE is applied before SE. This may be partly explained by the role of cytoskeletal desmin and alpha-crystallin B proteins, which are known for their protection function of the myofiber integrity and cellular stabilization [85]. In fact, it was reported that particularly eccentric exercise stimuli induce an increase in alpha-crystallin B phosphorylation [86]. In consequence, we hypothesise that generally trained individuals may have higher total amounts of alpha-crystallin B, owing to training-induced skeletal muscle adaptation, which then, in turn, protects these individuals from skeletal muscle fatigue induced by eccentric exercise stimuli. The observed results can also be explained by methodological factors. While cycling exercise results in specific local fatigue with substrate depletion of the knee extensors, running exercise is a more general exercise type and involves larger muscle groups. Given that all included studies applied knee extensor strength tests after the AE protocol, it can be speculated that cycling compared with running exercise resulted in larger performance decrements due to more pronounced local muscular fatigue of the knee extensors. This is supported by Dingwell et al. [87], who reported significant local muscle fatigue in highly trained cyclists after cycling at 100% of V̇O_2max_ until voluntary exhaustion. The authors detected significantly lower electromyographic median frequencies immediately after the cycling exercise, most prominent in the biceps femoris and the gastrocnemius.

## Limitations

This systematic review with meta-analysis has some limitations that warrant discussion. Above all, our results are valid for trained male adults. Thus, our findings cannot be translated to youth or female individuals. Another limitation is the heterogeneity of the endurance training protocols within the included studies. The various types of modalities, intensities, and durations demand different metabolic processes [88]. More specifically, intermittent vs continuous AE protocols may induce specific physiological adaptations. However, because of the limited number of available studies, protocol-specific analyses were not possible. Further, it can be anticipated that without an additional collection of physiological data and more structured modification of study designs and parameters (e.g., gene expression, cell signaling, blood markers of metabolism, and immunological stress) used in CT protocols concerning acute effects of AE on muscle strength and power, underlying (biological) mechanisms can hardly be identified. Studies involving trained individuals, which link performance with physiological data, are lacking and should be addressed in future studies. Moreover, the individual training status has a significant influence on short-term and long-term response to a specific training stimulus [89]. Especially in the context of CT, it is a challenge to describe individual performance capabilities [39]. The training status described in CT studies can refer to endurance activity (e.g., cycling), strength or power activity (e.g., long-jump), or both (e.g., rugby). In general, the transferability of the results must be verified on an individual basis because the group of subjects examined within our work is rather heterogeneous.

## Conclusions

The main findings of this systematic literature review with meta-analysis showed that AE results in significant and moderate declines in muscle strength but not power of involved muscles in trained male individuals. These results provide evidence of “acute interference” effects when AE is performed prior to SE. Further, our findings showed a negative influence of prior moderate-to-high intensity and longer duration AE on measures of muscle strength. Low-intensity and shorter duration AE did not mitigate strength performance. However, given the lack of statistically significant differences between subgroups (i.e., AE intensity [low vs moderate to high] and AE duration [≤ 30 min vs > 30 min]), it is not possible to conclude whether the acute AE effects on muscle strength were intensity and/or duration dependent. Moreover, our results suggest that running compared with cycling exercise results in diminished acute negative effects in trained individuals. These findings may guide practitioners to better prescribe single training sessions in which trained individuals have to apply AE before SE because of environmental and/or infrastructural reasons (e.g., availability of training facilities). Further original research is needed with female individuals and youth athletes. Finally, future studies should assess objective physiological markers (e.g., gene expression, cell signalling, blood markers of metabolism, and immunological stress) in addition to performance measures to gain more insight into the physiological aspects of AE-induced acute performance declines in muscle strength.

## Declarations

### Availability of data and material

The R script is available in Appendix S1 of the Electronic Supplementary Material [ESM]. Data from the included studies are available in the Appendix S2 of the ESM.
